# Efficacy of niclosamide and ivermectin suspension preparations in sheep parasitoses

**DOI:** 10.5455/javar.2024.k863

**Published:** 2024-12-29

**Authors:** Victor A. Marchenko, Yury A. Vasilenko, Ivan V. Biryukov, Marat S. Khalikov, Salavat S. Khalikov, Alireza Sazmand

**Affiliations:** 1 Gorno-Altay Research Institute of Agriculture (branch) of National Research, Tomsk State University, Barnaul, Russia; 2 A.N. Nesmeyanov Institute of Organoelement Compounds, Russian Academy of Science (RAS), Moscow, Russia; 3 Department of Pathobiology, Faculty of Veterinary Medicine, Bu-Ali Sina University, Hamedan, Iran

**Keywords:** Drug testing, helminth, mechanochemistry, ovine, parasiticidal activity

## Abstract

**Objective:**

This study aimed to examine the efficiency of newly formulated drugs based on mechanochemical modification of two widely used anthelmintic substances in suspension formulations against the main classes of helminths and parasitic arthropods.

**Materials and Methods:**

Solid-phase co-grinding of two substances, i.e., ivermectin (IM) and niclosamide (NS), with licorice extract and sodium succinate was performed in liquid-phase processing to obtain suspension formulations. Drug preparations were administered to the sheep of the experimental groups (5 experimental groups of 8 heads) with different concentrations of active substances, i.e., 3.0, 5.0, and 10.0 mg/kg of body weight (BW) NS and 0.2-mg/kg BW IM. Aqueous suspensions of the original substances in dosages of 0.2-mg/kg BW IM, 10.0-mg/kg NS, and placebo were included as controls. Efficacy of the formulations against gastrointestinal strongyles, *Trichuris* spp., *Moniezia* spp., and nasal bot larvae (*Oestrus ovis*) was assessed in naturally infected sheep by the number of surviving adult parasites post-treatment in the gastrointestinal tract, nasal passages, and sinuses.

**Results:**

Formulated suspensions showed an increased solubility of 5.5–13.1 times compared to the original substances. Treatment of sheep with the SusIN-10 drug containing 0.2-mg/kg BW IM and 10.0-mg/kg NS showed 100% efficiency against gastrointestinal strongyles, *Moniezia, *and nasal bot larvae. Formulations containing 0.2-mg/kg BW IM and 3.0- and 0.5-mg/kg NS could eliminate all of the *Trichuris* worms. Administration of original substances of IM and NS with recommended dosages did not result in the adult parasites’ complete elimination.

**Conclusion:**

Modification of the anthelmintic substances through mechanochemical methods made it possible to create formulations with a targeted spectrum of action, significantly higher water solubility, and optimal parasiticidal activity.

## Introduction

Parasitic diseases are major constraints in sheep farming worldwide. Considering the prevalence of mixed infections with different parasite taxa, the administration of complex antiparasitic preparations with a wide spectrum of activity is necessary. The use of multi-taxa-affecting drugs based on different substances makes it possible to affect the entire spectrum of parasites with a reduced volume of drug use and reduced frequency of animals’ manipulations [1]. It has been shown that most antiparasitic substances are organic compounds poorly soluble in water [2]; hence, to achieve the desired therapeutic effect, it is always necessary to significantly overestimate the dosage of the substance in the preparation, which leads to an increase in the product cost and, importantly, the release of higher amounts of the unchanged drugs and their metabolites into the environment [3].

To improve the solubility of antiparasitic substances and their pharmacokinetic properties, and also increase the effectiveness of various physicochemical methods, such as reducing the size of crystalline particles to nano-size by grinding and obtaining dosage forms such as nanosuspensions, microcapsules, etc. [4], preparing solid lipid nanoparticles and nanocapsules [5,6], designing polymeric forms of drugs in the form of micelles [7], and forming solid dispersions (SDs) of medicinal substances [8] are used. The compositions obtained with these methods will result in higher efficiency treatments by overcoming the disadvantages of low bioavailability, poor cell permeability, nonspecific distribution, and rapid elimination of antiparasitic drugs from the body [9].

Previously, we showed that modification of fenbendazole (FBZ)—a broad-spectrum dewormer that is used to treat many intestinal parasites—by joint grinding with licorice extract (LE) and sodium dioctyl sulfosuccinate (Na-DSS) resulted in high efficacy of obtained compositions [10], and we believe that the solubility of niclosamide (NS)—an anthelmintic medication used to treat tapeworm infections—will increase the same way. Licorice is a plant containing 25% glycyrrhizic acid (GA) and is known to have a wide range of biological activities, which helps to improve the membrane conductivity of medicinal substances [11]. Na-DSS, on the other hand, is used as an emulsifier in various technologies and has a pronounced antihypoxic and antioxidant effect [12].

Internal parasites complex of sheep in the farms of the region is represented by all the main taxa with fairly constant infection rates: nematodes (60%–80%), trematodes (20%–40%), cestodes (15%–25%), and botfly (65%–75%). In particular, 84%–94% of the sheep raised in this territory (Ust-Koksinsky region) were previously reported to be infected by *Oestrus ovis* larvae in their nasal passages and sinuses [13]. Therefore, it is advisable to conduct research not only on the synthesis and search for new anthelmintic substances, which is an extremely expensive path, but also on the modification of existing substances and the production of complex preparations from several substances with different mechanisms of action.

The aim of this study was to obtain complex therapeutic drugs based on substances of ivermectin (IM) and NS and test their parasiticidal activity against common parasitic infections of sheep, i.e., nematodes and cestodes of the gastrointestinal tract and larvae of the sheep botfly *O. ovis *(Linnaeus 1758) in the Altai Mountains.

The objective of this study was to examine the efficiency of newly formulated drugs based on the mechanochemical modification of two widely used anthelmintic substances, IM and NS, in suspension formulations against the main classes of helminths and parasitic arthropods.

## Materials and Methods

### Ethical approval

This study was approved by the State order of the Federal State Scientific Institution (FASCA No. 0534-2021-0005) and the FASCA Bioethics Commission, order No. 210, dated October 12, 2022. All applicable international, national, and/or institutional guidelines for the care and use of animals were followed.

### Materials

IM substance was purchased from Shandong Qilu King-Phar Pharmaceutical Co. Ltd. (Shandong, China). NS substance was obtained from Ghangzhou Yabang-Qh Pharmachem Co., Ltd. (Ghangzhou, China). LE—a dry, fine powder of dark brown color with a content of 25% GA—was taken from Visterra Ltd. (Altay, Russia). Na-DSS was purchased from Acras Organics (New Jersey, USA). Sodium salt of carboxymethyl cellulose (Na-CMC = blanose) was purchased from СP Kelco (Aanekoski, Finland).

### The target compositions in the form of aqueous suspensions were obtained as follows

First, the solid-phase mechanochemical treatment of the IM and NS substances was carried out with LE (stage 1) and further addition of Na-DSS (stage 2) under the conditions described earlier [16]. The obtained SD compositions IM:LE (1:9) and NS:LE (1:9), which are light brown powders with increased solubility of substances (Table 1), were subjected to further mechanical processing by adding the appropriate amount of Na-DSS to obtain the compositions IM:LE:Na-DSS (1.0:8.8:0.2) and NS:LE:Na-DSS (1.0:8.8:0.2). The products of mechanical processing were SD, which had an increased solubility (Table 1). Obtained SD were used to obtain the corresponding single suspension formulations of IM (SusI), containing 2.9% IM, and NS (SusN), containing 3.4% NS. Afterward, complex suspension formulations were prepared from single suspensions previously obtained, taking into account the required dosage of drugs. Three suspensions with the abbreviation Suspension of Ivermectin and Niclosamide (SusIN) were achieved, i.e., SusIN-3 (at the rate of 0.23 mg of IM and 3.51 mg of NS in 1 ml of suspension), SusIN-5 (at the rate of 0.23 mg of IM and 5.85 mg of NS in 1 ml of suspension), and SusIN-10 (at the rate of 0.23 mg of IM and 11.7 mg of NS in 1 ml of suspension).

To administer as controls, suspension samples of the initial substances (without LE and Na-DSS) were prepared by suspending IM and NS separately using a rotary stirrer in a 0.2% aqueous polymeric solution of Na-CMC at the rate of IM: 0.23 mg and NS: 11.7 mg in 1 ml of suspension.

**Table 1.** Solubility in the water of SD samples based on IM and NS.

Sample, its composition, preparation conditions, and content of IM and NS	Water solubility	
	Absolute (mg/l)	Increased (times)
IM (initial substance), 97.5% IM	4.0	−
SD of the IM:LE (1:9) after 3 h of mechanical treatment; 10.0% IM	21.9	5.5
SD of the IM:LE:Na-DSS (1.0:8.8:0.2) after 3 h of mechanochemical treatment; 10.0% IM	52.4	13.1
NS (initial substance), 98.0% NS	5.0	−
SD of the NS:LE (1:9) after 3 h of mechanochemical treatment; 10.0% NS	6.5	1.5
SD of the NS:LE:Na-DSS (1.0:8.8:0.2) after 3 h of mechanochemical treatment 10.0% NS	31.3	6.3

### Solubility study

The solubility of the resulting SD was determined by the amount of IM and NS in the filtrate after stirring samples of the SD in water for 3 h by high-performance liquid chromatography on an Agilent 1,200 chromatograph with a Zorbax Eclipse XDB-C18 column, 4.6 × 50 mm (Agilent Technologies, CA, USA); column temperature +30°C; diode-matrix detector. An acetonitrile acetate buffer pH 3.4 (55:45) was used as an eluent, the flow rate was 1 ml/min, and the sample volume was 5 µl [14]. The analysis error was ±3%.

### Determination of particle sizes in solutions of the suspension formulations

Dynamic light scattering (DLS) technology [15] was used to estimate the average particle size and polydispersity index of obtained suspension formulations by the Photocor Complex Instrument (Photocor, Moscow) at 25°C. The compositions were dissolved in distilled water before measurement. The results were obtained by measuring three times and taking the average value.

### Study animals

Previous studies in the Altai Mountains reported the maximum infection of helminths and *O. ovis* larvae to occur between September and October, during which antiparasitic treatments of sheep are recommended [1]. Henceforth, we chose this optimal period for assessing the effectiveness of the drugs.

A randomized and placebo-controlled study was carried out in October 2022 on a flock of sheep from the agricultural production cooperative “Amur” in the Ust-Koksinsky district of the Altai Republic in accordance with the Guidelines for the Experimental (Preclinical) Study of New Pharmacological Substances [16] and the European Convention for the Protection of Vertebrate Animals Used for Experimental and Other Scientific Purposes [17]. During the experiment, the sheep did not graze on the pasture; they were kept indoors and fed according to the norms and rations for feeding livestock [18].

Fifty sheep of the Gorno-Altai breed aged 16–18 months weighing 35–40 kg were included. Three days before the experiment, individual rectal fecal samples from 20 randomly selected animals were examined according to Kotelnikov–Khrenov’s method using a VIGIS counting chamber. Results showed infection with gastrointestinal strongyles in 55.0% (mean EPG = 75.2), *Trichuris* in 30.0% (mean EPG = 32.5), and *Moniezia* in 20.0% (mean EPG = 41.3) of animals. The relatively low infection rate is most likely due to the grazing of animals in the summer on high alpine pastures. Five experimental groups of 8 sheep and 1 control group of 10 sheep with close infection rates were randomly formed.

### Treatments of animals

Suspension preparations (SusIN) were fed to the sheep of the experimental groups at a dosage according to the NS—3.0, 5.0, and 10.0 mg/kg body weight (BW) and IM at 0.2-mg/kg BW. Aqueous suspensions of the original substances were used in dosages of IM—0.2-mg/kg BW, NS—10.0-mg/kg BW, and placebo (control group) in the form of 0.2% blanose were also fed to animals with the same volume as SusINs.

For assessment of possible side effects, clinical parameters, such as temperature, pulse rate, respiration rate, rumen movement, and behavior, were checked before and on days 1, 3, and 5 post-treatment in the morning before feeding according to the method of veterinary clinical laboratory diagnostics [19].

### Study of the parasitical activity of drugs

Anthelmintic efficacy of the treatments was assessed 10 days post-treatment after slaughter at a local meat processing plant by naked-eye examination of the abomasum, small, and large intestines. At the same time, the mucous membranes of the nasal and adnexal cavities of the sheep heads were examined for the presence of botfly larvae. The proportion of infected animals, arithmetic and geometric mean parasite counts, and taxonomic identification of the recovered parasites were recorded [20].

Parasiticidal activity evaluation based on the calculation of two effectiveness indicators as used previously [1]: IE % = decrease in the arithmetic mean number of parasites of the experimental groups in relation to the control and EF % = decrease in the geometric mean number of parasites of the experimental groups in relation to the control.

To compare the differences between the experimental and control groups of animals, a *t*-test was used in SAS/Stat software (SAS version No. 9, System for Windows). *p*-values < 0.05 were considered statistically significant differences. The use of two methods for calculating the effectiveness (EF and IE) allows you to more fully judge the parasiticidal activity of drugs.

## Results

### Analysis of the solubility of SD

The solubility of mechanochemically modified formulations containing IM and NS increased 5.5 and 1.5 times after co-grinding of LE with the original substance. Additionally, after the addition of Na-DSS to the SDs, the solubilities increased by a factor of 13.1 and 6.3. Hence, two compositions, IM:LE:Na-DSS (1:8.8:0.2) and NS:LE:Na-DSS (1:8.8:0.2), were chosen for biological testing (Table 1).

### Preparation of suspension formulations

Samples of aqueous suspensions of SusIN were prepared from previously obtained SD by suspending them with a rotary mixer and loading the calculated amounts of these SDs. The corresponding SusIN suspensions were obtained and presented in the calculated volume per kg of sheep BW as follows: i) 0.83 ml of SusIN-3 suspension contained 0.2-mg IM and 3.0-mg NS; ii) 0.83 ml of SusIN-5 suspension contained 0.2-mg IM and 5.0-mg NS; and iii) 0.83 ml of SusIN-10 suspension contained 0.2-mg IM and 10.0-mg NS.

**Figure 1.**
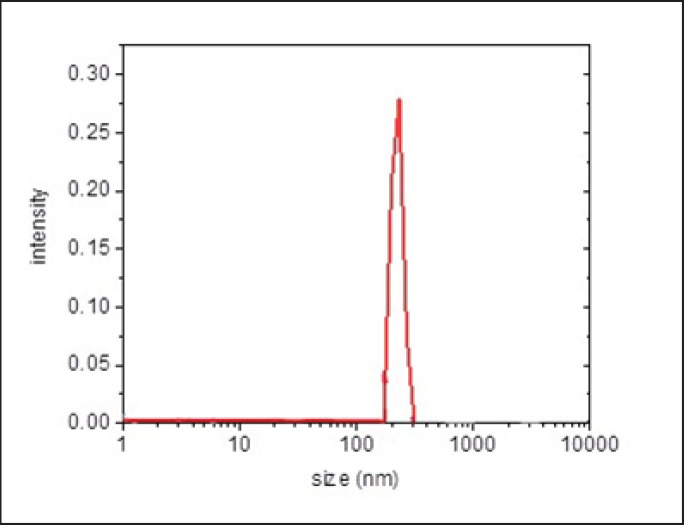
DLS measurement of particle size of aqueous suspension of SusIN-10.

The particle size of the resulting suspensions was determined as 225 ± 40 nm according to the DLS technology [15] using Photocor Compact-Z (Fotokor, Moscow, Russia). The size distribution of the particles in the aqueous suspension of SusIN-10 is shown in Figure 1.

According to DLS results, a narrow monodisperse size distribution (225 ± 40 nm) was observed for nanosuspension particles based on LE and Na-DSS. It can be assumed that these are the micelles of GA, the content of which in LE is about 25%. This is consistent with previous studies on the sizes of GA micelles [21].

### Clinical data

Examined health parameters of the animals remained within the physiological range.

### Parasitological data

Parasiticidal activity of drugs in the case of the gastrointestinal tract strongyles is shown in Table 2. When examining the gastrointestinal tracts of sheep in the experimental and control groups, the adult stages of *Teladorsagia*,* Trichostrongylus*,* Nematodirus*, *Oesophagostomum*, and* Chabertia *were identified. Due to the low number of surviving adult parasites in the experimental groups, calculating the drug efficacy against specific parasite genera was not feasible. Therefore, the total number of infected animals and the total count of all adult helminths of the order Strongylida were used for the efficacy calculations. In experimental groups 2 (SusIN-3) and 4 (SusIN-10), all performance indicators were 100%. The effectiveness of the SusIN-5 suspension (group 3) was also quite high, as an IE% of 97.4 and an Ef% of 92.3 were recorded. Although the original form of IM (group 5) showed effectiveness, the efficacy indicators were lower than those of all three SusIN-3, SusIN-5, and SusIN-10 groups. As expected, administration of the original substance of NS (group 6) at a dosage of 10-mg/kg BW was not effective.

**Table 2.** Efficiency of samples of suspensions in gastrointestinal strongylosis of sheep.

Experiment number/group of animals	# in group	Test product	Dose (mg of active substance/kg BW)	# remaining parasites^a^	IE^c^ %	Ef^d^ %	*p*-value^e^
1/Control	10	Placebo	0	19.1 ± 6.8 1.3 ± 0.17	-	-	-
1/Treatment	8	SusIN-3	Modified IM 0.2 Modified NS 3.0	0 0	100	100	NA^f^
2/Treatment	8	SusIN-5	Modified IM 0.2 Modified NS 5.0	0.5 ± 0.27 0.1 ± 0.08	97.4	92.3	<0.01
3/Treatment	8	SusIN-10	Modified IM 0.2 Modified NS 10.0	0 0	100	100	NA
4/Treatment	8	SusIM	Original substance IM 0.2	1.3 ± 0.5 0.34 ± 0.13	93.2	73.9	<0.01
5/Treatment	8	SusNS	Original substance NS 10.0	17.8 ± 5.6 1.1 ± 0.15	6.8	15.4	>0.05

^a^Number of parasites—the numerator is the arithmetic mean and the denominator is the geometric mean number of parasites; ^b^EE: extensive efficiency, the proportion of animals freed from parasites in relation to the control; ^c^IE: intensity efficiency, decrease in the average (arithmetic) indicator of the number of eggs in relation to control; ^d^Ef: decrease in the geometric mean values of the number of helminth eggs of the experimental groups in relation to the control; ^e ^statistically significant at *p* ≤ 0.05 when geometric means were compared to placebo;^ f ^NA: statistical analysis was not performed.

**Table 3.** Efficacy of suspension samples in sheep trichurosis.

Experiment number/group of animals	# in group	Test product	Dose (mg of active substance/kg BW)	# remaining parasites^a^	IE^c^ %	Ef^d^ %	*p*-value^e^
1/Control	10	Placebo	0	6.2 ± 2.8 1.16 ± 0.09	-	-	-
1/Treatment	8	SusIN-3	Modified IM 0.2 Modified NS 3.0	0 0	100	100	NA^f^
2/Treatment	8	SusIN-5	Modified IM 0.2 Modified NS 5.0	0 0	100	100	NA
3/Treatment	8	SusIN-10	Modified IM 0.2 Modified NS 10.0	0.37 ± 0.26 0.1 ± 0.1	94.1	91.4	< 0.01
4/Treatment	8	SusIM	Original substance IM 0.2	1.12± 0.6 0.46 ± 0.09	82.0	60.4	< 0.05
5/Treatment	8	SusNS	Original substance NS 10.0	6.75 ± 3.2 1.04 ± 0.17	0.0	0.0	NA

^a-f^ For the abbreviations see footnotes of Table 2.

Inspection of the large intestines revealed that in experimental groups 2 (SusIN-3) and 3 (SusIN-5), all performance indicators were 100%. The effectiveness of the SusIN-10 suspension (group 4) was also good, i.e., both quite IE and Ef >91%, but unchanged IM (group 5) at the recommended dosage of 0.2-mg/kg BW showed lower efficacy (IE = 82.0% and Ef = 60.4%). Suspension of substance NS (group 6), at a dosage of 10-mg/kg BW with trichurosis, did not show parasiticidal activity (Table 3).

With regard to the cestocidal effect of the formulations, unchanged NS with a dose of 10 mg/kg BW, also SusIN-3 and SusIN-5, showed no or low efficiency. Suspension SusIN-10, however, proved to be effective in the removal of all adult tapeworms (Table 4).

When examining the mucous membranes of the nasal passages, ethmoid bones, and adnexal cavities of the sheep heads, 1st and 2nd instar larvae of *O. ovis* were found in all groups except animals receiving SusIN-5 and SusIN-10 samples (100% efficacy). The activity of SusIN-3 and the original substance of IM was also acceptable (Table 5).

**Table 4.** Efficacy of suspension samples in sheep monieziosis.

Experiment number/group of animals	# in group	Test product	Dose (mg of active substance/kg BW)	# remaining parasites^a^	IE^c^ %	Ef^d^ %	*p*-value^e^
1/Control	10	Placebo	0	0.9 ± 0.48 0.46 ± 0.09	-	-	-
1/Treatment	8	SusIN-3	Modified IM 0.2 Modified NS 3.0	1.0 ± 0.5 0.42 ± 0.06	0.0	0.0	NA^i^
2/Treatment	8	SusIN-5	Modified IM 0.2 Modified NS 5.0	0.62 ± 0.32 0.2 ± 0.1	31.2	56.5	> 0.05
3/Treatment	8	SusIN-10	Modified IM 0.2 Modified NS 10.0	0.0	100	100	NA
4/Treatment	8	SusIM	Original substance IM 0.2	0.87 ± 0.4 0.3 ± 0.12	3.4	34.8	> 0.05
5/Treatment	8	SusNS	Original substance NS 10.0	0.62 ± 0.42 0.39 ± 0.09	31.2	15.3	> 0.05

^a-f^ For the abbreviations, see the footnotes of Table 2.

**Table 5.** Efficacy of suspension samples in sheep estrosis.

Experiment number/group of animals	# in group	Test product	Dose (mg of active substance/kg BW)	# remaining parasites^a^	IE^c^ %	Ef^d^ %	*p*-value^e^
1/Control	10	Placebo	0	11.2 ± 2.8 1.18 ± 0.06	-	-	-
1/Treatment	8	SusIN-3	Modified IM 0.2 Modified NS 3.0	0.86 ± 0.49 0.26 ± 0.16	92.4	78.0	<0.05
2/Treatment	8	SusIN-5	Modified IM 0.2 Modified NS 5.0	0.0	100	100	NA^i^
3/Treatment	8	SusIN-10	Modified IM 0.2 Modified NS 10.0	0.0	100	100	NA
4/Treatment	8	SusIM	Original substance IM 0.2	1.13 ± 0.58 0.46 ± 0.09	89.9	61.1	<0.01
5/Treatment	8	SusNS	Original substance NS 10.0	10.6 ± 3.6 1.05 ± 0.14	5.4	11.1	>0.05

^a-f^ For the abbreviations, see the footnotes of Table 2.

## Discussion

The diverse taxonomic composition of the internal parasite complex in sheep, comprising nematodes, cestodes, trematodes, and nasopharyngeal myiasis, necessitates the use of broad-spectrum antiparasitic compounds for effective treatment. Therefore, in this study, we formulated mechanochemically modified NS and IM preparations by their joint grinding with LE and Na-DSS and showed that the outcome suspensions possess relatively better efficiency for the elimination of target parasites in comparison with original unchanged drugs given at recommended doses. This observation can be explained by increased water solubility of the suspension forms, i.e., by a factor of 13.1 for IM:LE:Na-DSS and 6.3 for NS:LE:Na-DSS compared to the initial substances, which leads to higher bioavailability and consequently antiparasitic activity [22,23].

In this regard, LE might have played an important role in increasing the solubility of the suspension forms, i.e., by a factor of 5.5 for IM:LE and 1.5 for NS:LE compared to the initial substances. Indeed, LE contains 25% of a naturally occurring metabolite, GA, which is a widely used medicinal component as an anti-inflammatory agent, antiulcer agent, anti-allergy agent, and anti-psoriatic agent [24]. GA has the ability to change the properties of cell membranes, even at micromolar concentrations, and it can provide new insight into the mechanism of enhancement of drug bioavailability in the presence of GA. Researchers suggest this substance as an effective drug carrier, which enhances the solubility of low-soluble drugs, as well as enhances their penetration through cell membranes [11]. Previous studies showed that using GA and its derivatives for the mechanochemical modification of various poorly soluble substances contributes not only to increasing their solubility but also to enhancing their biological efficiency. For instance, it has been reported that complexing praziquantel (PZQ), the most commonly used anthelmintic drug for treating trematodoses, with disodium glycyrrhizinate in the 1:10, ratio had higher bioavailability than PZQ substance and reduced the number of *Opisthorchis felineus* helminths in the liver by 87% [25,26]. In another study, joint mechanochemical treatment of PZQ with disodium salt of GA (Na_2_GA) led to significantly increased solubility by 3 times, reduction of particle sizes, amorphization of substance, incorporation with micelles of GA, and high cestodicidal efficacy.

The emulsifier Na-DSS with antihypoxic and antioxidant effects [12] was another substance we employed to increase the water solubility of the formulations. Previously, it was shown that modification of FBZ to FBZ:LE:Na-DSS led to increased solubility of FBZ up to 27 times, which contributed to an increase in its permeability through biological membranes and an increase in the activity of the drug [10]. The reason behind the higher anthelmintic action of FBZ:LE:Na-DSS was explained to be the smaller size of FBZ, loss of crystallinity, amorphization, and inclusion of its molecules on the surface and inside the pores of polymers; the increase in solubility and permeability through biological membranes [27].

As a limitation, in this study, we did not examine the parasitocidal effectiveness of the formulations against pulmonary nematodes, liver flukes, lancet flukes, and the sheep ked *Melophagus ovinus,* which are common and widespread. Future studies are needed to elucidate this issue. Such results were achieved using data known in the literature on these substances and their modification by various methods.

## Conclusion

This study demonstrated that the mechanochemical solid-phase modification of active pharmaceutical ingredients can lead to the development of new complex antiparasitic drugs with a broad spectrum of efficacy. Moreover, this approach can significantly reduce the required dose and frequency of treatment, thereby minimizing the negative impact on the animals and promoting higher standards of animal welfare. Furthermore, the preparation of drugs using this technology is waste-free and safe both for production and the environment [28].

## List of abbreviations

BW, body weight; DLS, dynamic light scattering; EE, extensive efficiency; Ef, decrease in the geometric mean values of the number of helminth eggs of the experimental groups in relation to the control; EPG, egg per gram of feces; FASCA, federal altai scientific center for agrobiotechnology; FBZ, fenbendazole; GA, Glycyrrhizic acid; IE, Intensity efficiency; IM, Ivermectin; LE, Licorice extract; Na-DSS, Sodium dioctylsulfosuccinate; Na-CMC, Sodium salt of carboxymethyl cellulose; NS, niclosamide; PZQ, praziquantel; SD, solid dispersion; SusIN; suspension of ivermectin and niclosamide.
